# Reducing Unplanned Extubations in a Level IV Neonatal Intensive Care Unit: The Elusive Benchmark

**DOI:** 10.1097/pq9.0000000000000337

**Published:** 2020-10-23

**Authors:** Maheshwar Mahaseth, Eunice Woldt, Mary Ellen Zajac, Brande Mazzeo, Jennie Basirico, Girija Natarajan

**Affiliations:** From the * Department of Pediatrics, Wayne State University, Detroit, Mich.; † Department of Pediatrics, Children’s Hospital of Michigan, Detroit, Mich.

## Abstract

Unplanned extubation (UE) is a common adverse event in the neonatal intensive care unit (NICU). At our level IV NICU, we initiated a quality improvement project in 2012 to reduce UE rates from 7.47 to below 100 intubated days. We describe the strategies used.

**Methods:**

Multiple plan-do-study-act cycles were performed to address key drivers. Important interventions focused on staff education, consistent use of a new endotracheal (ET) tube securing device, 2 providers during bedside activities, documentation of ET tube position, and targeted sedation. Process measures included immediate root cause analyses for UE events and the use of the endotracheal tube securing device. The primary outcome was the UE rate per 100 intubated days.

**Results:**

Over a nearly 6-year study period, quarterly UE rates decreased from 7.19 to 0.66 per 100 intubated days. The proportion of neonates requiring reintubation remained stable (64%–76%). Rates of root cause analysis completion and use of the ET securing device were more than 90% in the last 3 years of the study. The majority (61%) of UE events occurred in infants with birth weights greater than 2 kg, and 46% of infants had a prior UE. UE was associated with desaturation (50%), bradycardia (22%), and the need for resuscitation (7%).

**Conclusions:**

This quality improvement effort in a level IV NICU achieved a reduction in UE rates to below 1 per 100 intubated days after more than 5 years. Consistency in practices and widespread communication with the staff was critical to the effort.

## INTRODUCTION

Critically ill infants in the neonatal intensive care unit (NICU) often require endotracheal (ET) intubation and mechanical ventilation for prolonged periods. One complication associated with ET intubation is unplanned extubation (UE) or accidental early removal of ET tube by the patient or staff.^1,2^ UE requiring reintubation is the fourth most common adverse event in the NICU in North America after nosocomial infection, catheter infiltration, and abnormal cranial imaging.^3^ Neonates are more prone to UE than older children and adults, because of their prolonged duration of intubation, shorter trachea, uncuffed ET tube, and possibly, less sedation.^2^ The incidence of UE across NICUs varies from 0.14 to 5.3 per 100 patient intubated days, with the variation attributed to differences in NICU size, level of care, staffing and patient acuity, ET tube securing methods, and sedation practices.^4^ Bedside activities such as weighing, suctioning, and kangaroo care and procedures such as blood draw, line placement, and transport may be the immediate antecedents of a UE.^4^ UE is associated with rapid deterioration in cardiorespiratory status, need for resuscitation, airway trauma, subglottic stenosis, prolonged ventilation, prolonged hospital stay, and increased infection.^5^

A systematic review of 13 studies in adult ICUs showed that continuous quality improvement (CQI) programs effectively reduced UE rates between 0.11 and 2.72 per 100 intubation days.^6^ Interventions included standardization of procedures, staff education, surveillance, and identification and management of high-risk patients.^6^ In the pediatric intensive care unit, a meta-analysis of 5 quality improvement (QI) studies reported a mean incidence of UE of 1.19 (95% CI: 0.89−1.49).^5,7–12^ Key interventions were targeted at caregivers, bedside procedures, management of patient agitation, and ET tube care.^5,7–12^ All the QI studies were for 12 (n = 4) or 24 (n = 1) months duration, and 3 showed UE rate reductions ranging from 0.29 to 2.59 events per 100 intubated days. Many published reports of QI initiatives in the NICU have demonstrated a reduction in UE rates over short periods.^2,13–17^ Sustaining the accepted benchmark UE rate below 1 per 100 intubated days has remained elusive.^2,13–17^

At our level IV NICU, we initiated a CQI project in November 2012 to reduce UE rates after our multidisciplinary safety team identified a high UE rate. We aimed to reduce the incidence of UE from a baseline of 7.34 to below 1 per 100 intubated days in the next 12 months. In the current study, we describe our strategies and assess whether a CQI project over 5 years continues to sustain gains.

## METHODS

### Setting

We conducted the current CQI initiative at an academic level IV 40-bed NICU at Children’s Hospital of Michigan. The NICU was a pod-design unit with 6 beds in each pod until June 2017; we then changed to a single-room design NICU. The entirely outborn NICU is a referral center with extracorporeal membrane oxygenation capabilities. Infants were transferred from other NICUs or readmitted from the emergency room after discharge if they met criteria for age (below 1 month overall and less than 6 months if discharged from NICU) and diagnosis. The NICU staff includes 5 to 7 residents typically rotating each block during weekdays and 3 on weekends, 2 neonatal-perinatal medicine fellows during weekdays and 1 on weekends, 1 to 2 neonatal nurse practitioners, and 2 board-certified neonatologists. The pediatric surgery team, including trainees, managed infants with surgical conditions. NICU-dedicated respiratory therapists, pharmacists, dieticians, and occupational/physical therapists are other members of the patient care team.

The nurse to patient ratio was determined by patient acuity and was 1:1 or 1:2 for intubated infants. All patients were orally intubated, and we did not use physical restraints. Continuous infusions of sedative or analgesic medications were used as needed for optimizing ventilation. The most common medications used were midazolam (starting dose 0.05 mg/kg/h) and fentanyl (starting dose 1 μg/kg/h), with occasional morphine. Single doses of neuromuscular blockers were used on rare occasions when we could not achieve synchronous ventilation despite sedation. Before the QI study, ET tubes were secured using 2 pieces of tape cut in Y shape, anchored to the infant’s face.

The Wayne State University Institutional Review Board determined that this was a QI project and not human subject research. Therefore, IRB review and approval was not required.

### Interventions

Our multidisciplinary safety team of physicians (fellows and attending physicians), nurses, Clinical Nurse Specialist (NICU Quality Leader), and respiratory therapists studied our baseline rates of UEs, reviewed the available literature, and analyzed key drivers.

### Key Drivers

**Fig. 1.**
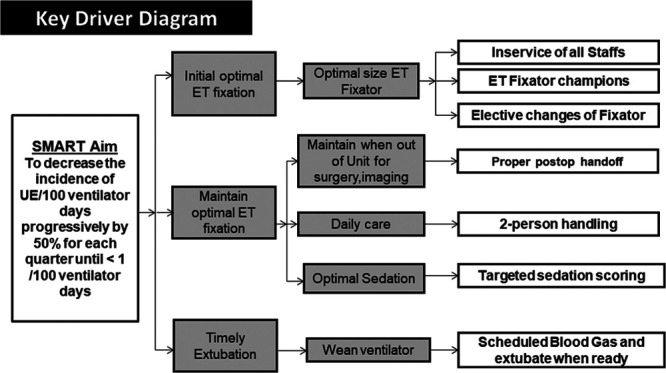
Key drivers of the quality improvement project. SMART, specific, measurable, attainable, relevant, timely.

Identified key drivers (Fig. 1) included optimal initial ET tube fixation, appropriate ongoing ET tube care, and timely extubation. Intervention steps included (1) an in-service for staff on UE rates and associated risks; (2) consistent, standardized use of an ET tube securing device in all patients and ET fixator champions (who trained staff, provided feedback on size and technique to staff, and audited correct use); (2) targeting high-risk situations, such as bedside procedures, postoperative period, and transport in and out of the unit to develop standardized operations; (4) monitoring sedation levels using a tool; and (5) communication of patient-specific sedation goals and prompt weaning and extubation when medically ready.

### Plan-Do-Study-Act Cycles

We targeted these key drivers with multiple plan-do-study-act improvement cycles including (1) manikin-based demonstration and tip sheet for appropriate placement of securing device (NeoBar: Neotech, Valencia, CA); (2) documentation of ET placement with each nursing assessment and ventilator check; (3) immediate root cause analyses after UEs, including nursing, respiratory therapists, and physicians; (4) 2-person handling during all tube manipulations and bedside procedures; (5) traffic stop signs (red octagons) at the bedside for infants with a prior UE with the reminder for physicians to call the nurse before examining the infant; (6) perioperative handoff tool to include discussion of the airway; and (7) consistent methods to position and support ventilator tubing.

Key interventions focused on staff education, consistent use of the new ET tube securing device, 2 providers during bedside activities, documentation of ET tube position, and targeted sedation. The timeline for the interventions is shown in Table 1. We performed random audits during certain PDSA cycles in infants for whom the securing device was used, and compliance with 2-person handling and ET tube documentation was evaluated. We conducted written audits on the use of the perioperative written handoff tools and whether there were any perioperative respiratory care failures (defined as adverse respiratory events within 24 hours of a surgical procedure such as the unintended need for intubation after extubation or pneumothorax) in the last quarter of 2014 and first quarter of 2015. The bedside nurse completed the UE apparent cause analysis (ACA) with details on staffing assignment, patient characteristics, antecedent procedures within 15 minutes before the event, whether sedation (as needed or as an infusion) was ordered, description of and effects of UE (starting 2016), and etiology of UE, in the opinion of the present staff (physician and respiratory therapist). The clinical nurse specialist reviewed the ACA and provided feedback on the optimal care of the ET tube.

**Table 1.** Key Interventions with Timeline through the Study Period

Key Interventions	Timeline for Initiation
UE data collection and display	April 2013
ET tube fixator standardization, mannequin training, and in-service of staff	April 2013; April to May 2017, education of all staff with quiz
RCA performance	May 2013; form modified January 2016
2-person handling, ET tube position documentation by nurse q 4 hours	February to April 2014
N-PASS sedation scoring education, target sedation level, and sedation prn	June 2014
ET tube position documentation by respiratory therapist q 4 hours	February to April 2015
Weaning and prompt extubation when medically ready	May 2015

RCA, root cause analysis; q, every; prn, as needed.

In April and May 2017, 100% of nurses completed a quiz and reviewed ET placement tips with a repeat hands-on demonstration of ET securement on a manikin. We collected data on UE in real time and prominently displayed UE events at several locations in the NICU. UEs were discussed with all providers in the daily huddle and with hospital management monthly. The Neonatal Pain, Agitation and Sedation Scale (N-PASS) was introduced for all intubated infants once a shift, with discussion on rounds of a target sedation level, based on the infant’s respiratory support, condition, and risk for UE.^18^ The quantitative assessment of sedation level using N-PASS has been shown to correlate with the bedside judgment of need for alteration of therapy for pain and sedation.^19^ The N-PASS uses 5 criteria: crying/irritability, behavioral state, facial expression, extremity tone, and vital signs. The sedation score has a range of 0 to 10, with 0 to 2 possible points for each criterion. We completed N-PASS education in 2014. Staff were required to discuss the correct use of the sedation scale and pass the quiz with a score of more than 80%.

### Measures

We calculated the primary outcome, UE rate, as the number of events divided by the number of intubated days, multiplied by 100 on all admitted infants with an ET tube. We excluded infants with tracheostomies or UE events in which ET tube was replaced (typically by a larger tube) despite correct placement demonstrated by capnography, chest rise, or auscultation. The nurse in charge of each shift kept count of UE events and reviewed daily by the clinical nurse specialist. Secondary outcomes included the number of UE events that resulted in a reintubation within 4 hours, the proportion of UE events that required cardiopulmonary resuscitation, or events associated with desaturations or bradycardia. Rates of the UE ACA completion and ET securing device use were process measures. We categorized the etiology of UE documented in the ACA as patient related (cough, secretions or emesis), procedures related (imaging, lab draws), endotracheal tube securement related, practice-related (2-person handling, communication errors), or multiple. We tracked doses of sedative used (obtained from the pharmacy as a report) as a balancing measure.

### Statistical Analysis

We used statistical process control U charts to analyze changes in the outcome measures over time. Special cause variation was defined according to statistical process control rules.^20^ Characteristics associated with UE were compared over the years using the chi-square test. We performed statistical analyses using SPSS version 22.0 (IBM Corporation).

## RESULTS

**Fig. 2.**
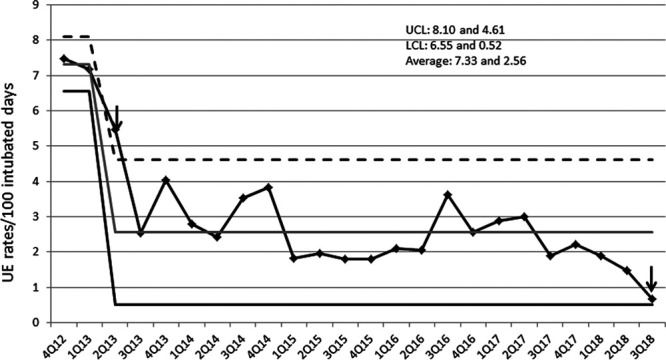
U chart with UE/100 intubated days for each quarter through the study period. UCL, upper control limit; LCL, lower control limit.

Table 2 describes baseline characteristics, process, and outcome measures over the years of the CQI project. The annual rates of admissions ranged from 732 to 997, and a quarter to third of the patient-days were ventilated days. ACAs were completed in more than 90% of UE events throughout, and the ET securing device was used more than 90% in the last 3 years. The rates of UE/100 ventilated days declined from baseline initially in the third quarter of 2013 and remained stable until the second quarter of 2017(Fig. 2). We noted trends of reduction in rates in the last 5 quarters of data collection. The benchmark UE rate below 1 was achieved in 5 of 9 months (last 4 consecutive months) in 2018. UE quarterly rates decreased from 7.19 in the first quarter of 2013 to 0.66 per 100 intubated days in the third quarter of 2018. Figure 2 is a U chart depicting this change and meeting criterion for special cause variation. The proportion of neonates requiring reintubation ranged from 64% to 76%, with the UE requiring reintubation rate in the last quarter being 0.50 per 100 intubated days. Sedative/analgesic (“as-needed” doses) used over the years also remained stable. In 2014, 105 of 112 (93.8%) nurses completed the N-PASS education successfully. The perioperative handoff tool audits (10 random surgeries each month from November 2014 through December 2015) revealed 100% compliance (n = 25) in 2014 and 91.2% (N = 114) use in 2015, without any respiratory care failures.

**Table 2.** Description of Baseline NICU Characteristics, Process, Outcome, and Balancing Measures

	2012	2013	2014	2015	2016	2017	2018 (January to September)
Baseline NICU characteristics							
NICU Admissions (n)	766	958	977	997	732	773	586
Patient-days	9,945	10,287	11,617	11,999	10,232	11,943	9,392
Ventilator days	3,481	2,783	3,073	3,048	3,272	2,156	2,065
% ventilated/patient-days	35%	27%	34%	27%	32%	24%	22%
Process measures							
RCA completed %	—	100%	100%	98%	93%	98%	100%
NeoBar used %	—	64%	72%	73%	90%	90%	90%
Outcome measures							
UE numbers	34	129	96	56	82	69	29
UE/100 intubated days	7.47	4.64	3.12	1.84	2.51	2.50	1.40
Reintubation needed %	—	68%	71%	70%	76%	64%	69%
UE requiring reintubation rate		3.09	2.21	1.28	1.90	1.62	0.97
Balancing measures							
Sedative/analgesic doses	—	—	57,724	58,113	59,677	49,785	36,310
Fentanyl doses	—	—	32,422	32,716	33,641	28,163	20,169
Midazolam doses	—	—	25,302	25,397	26,036	21,622	16,141

Table 3 describes characteristics associated with UE and their short-term adverse effects. The proportion of UE events during the day shift increased over time, as did the use of the securing device and 1:1 staffing. The majority (61%) of UE events occurred in infants with birth weights more than 2 kg; 46% of infants had a prior UE. Most (95%) of infants were on a conventional ventilator, and 53% were in the acute phase of respiratory distress at the time of UE; only 10% of UE occurred in babies who were extubation ready. UE was commonly (50%) associated with desaturation, less often (22%) with bradycardia, and rarely (7%) resuscitation was needed.

**Table 3.** Characteristics Associated with UE and Their Short-Term Adverse Effects

Characteristics n (%)	2013 (n = 108)	2014 (n = 94)	2015 (n = 55)	2016 (n = 82)	2017 (n = 67)	2018 (n = 29)	*P*
Day shift	47/102 (46%)	38 (40%)	22 (40%)	39 (48%)	41 (61%)	16 (55%)	0.006
Birth weight (kg)							0.005
<1	12 (12%)	11 (12%)	11 (22%)	17 (23%)	14 (20%)	1 (4%)	
1.001−2	25 (26%)	26 (28%)	6 (12%)	17 (23%)	12 (18%)	9 (31%)	
2.001−3	25 (26%)	36 (38%)	16 (31%)	11 (15%)	19 (28%)	5 (17%)	
>3	36 (37%)	21 (22%)	18 (35%)	28 (38%)	22 (33%)	14 (48%)	
Prior extubation	—	—	—	32/73 (44%)	30/67 (45%)	15/29 (52%)	0.730
SIMV	—	—	—	69/74(93%)	65/67(97%)	19 (66%)	0.538
Acute phase				42 (58%)	28 (42%)	7 (24%)	0.215
Weaning				22 (31%)	30 (45%)	3 (10%)	
Ext-ready				8 (11%)	9 (13%)		
NeoBar used	65/101 (64%)	68/94(72%	37/51 (73%)	64/71 (90%)	60/67 (90%)	26/29(90%)	0.003
Correct	103/108 (95%)			62/66 (94%)	56/62 (90%)	19/21 (91%)	0.728
Secured	79/98 (81%)	82/94 (87%)	46/51 (90%)	57/66 (86%)	53/62 (85%)	19/21 (91%)	0.792
Excessive secretions	47/98 (48%)	36/94 (38%)	18/51 (35%)	25/70 (36%)	36/67 (54%)	13/29 (45%)	0.169
Sedation ordered	66/98 (67%)	69/94 (73%)	35/51 (69%)	65/75(87%)	61/67(91%)	20/29 (69%)	0.001
Staffing 1:1	11/73 (15%)	27/94 (29%)	5/51 (10%)	15/72 (21%)	23/67 (34%)	17/29 (59%)	0.001
Procedure before UE	28/100 (28%)	73/94 (78%)	28/51 (55%)	48/72 (67%)	44/65 (68%)	19/29 (65%)	0.001
Etiology of UE							0.010
Other				10 (14)	2 (3%)	0	
Patient related				20 (27%)	13 (19%)	10 (34%)	
Procedure				1 (1%)	6 (9%)	3 (10%)	
ET tube related				8 (11%)	6 (9%)	4 (14%)	
Practice related				15 (21%)	11 (17%)	8 (28%)	
Multiple				19 (26%)	29 (43%)	4 (14%)	
Bradycardia %	—			15/73 (21%)	12/67 (18%)	10/29 (34%)	0.184
Desaturation %	—			34/73 (47%)	39/67 (58%)	12/29 (41%)	0.223
BP change %	—			13/73 (18%)	13/67 (19%)	0	0.547
CPR required	—						
Chest compression				5 (6.9%)	4 (6%)	3 (10%)	0.741
Medications				1 (1.4%)	1 (1%)	3 (10%)	0.036

The denominator reflects the events in which the information was available and complete.

BP, blood pressure; CPR, cardiopulmonary resuscitation; Ext-ready, extubation ready; SIMV, synchronized intermittent mandatory ventilation.

## DISCUSSION

We report the results of a CQI project at a single level IV NICU, which included evidence-based better practices to reduce the rate of UE. We implemented consistent fixation procedures for the ET tube and attempted to address many of the risk factors with sedation orders and 2-person handling. The ACA was comprehensive and included data on all risk factors. Communication of UE event was immediate, and dissemination was wide; we believe that this led to increased awareness among staff early in the QI project.

During the CQI project, we implemented the N-PASS sedation scale and a perioperative discussion of the ET tube. From the initial high rates, there was a decrease in the UE rate 2 years after the initiation of the CQI project to below 2 per 100 intubated days. Over the subsequent 2 years, UE rates stayed stable. The benchmark rate of below 1 per 100 intubated days was achieved only in the latest quarter of 2018, 5 years into the CQI.

There are several reasons for the slow decline in UE rates. The securing device chosen has a steep learning curve, and appropriate size selection and placement required training and experience. During the CQI duration, our NICU had nursing attrition and staffing problems, which were managed by new hires, travel nurses, and a pool of nurses who rotated between the pediatric intensive care unit and NICU. Although these measures were innovative, buy-in to the CQI goal and adherence to some practices, chiefly, 2-person handling (based on ACAs and random audits), was not consistent. A move to a new single-room NICU in 2017 was also likely a distraction because a successful transition became the priority for the staff and leadership during that time.

A meta-analysis identified restlessness/agitation (13%−89%), self-extubation by the patient (62%), poor fixation (8.5%−31%), and manipulation of the ET tube (17%−30%) and procedure at bedside (27.5%−51%) as risk factors for UE in the NICU.^4^ Other reported contributing factors were previous UE (47%), weaning stage from mechanical ventilation (44.4%), day shift (51%), and prolonged intubation, with each day increasing UE risk by 3% (4). The etiology of UE in our study was more patient related and fewer ET tube or procedural triggers. There were statistically significant differences in UE events’ characteristics over the years, but the small numbers precluded any broader conclusions. The move to a single-room NICU was associated with a small change in the nursing staffing model starting in June 2017.

The limited previous CQI initiatives have included standardization of procedures and care practices to secure the ET tube, education, analgesia and sedation, and early extubation.^4^ Specific interventions included 2-person retaping and during any procedures, placement of airway alert cards at the bedsides, standardized fixation with a device, ACA of each event, daily display of events, and use of restraining mittens in infants older than 34 weeks postmenstrual age.^3^ Others have defined standards for head and airway position during chest radiograph and included discussion of the airway in the postoperative handoff.^17^ Rates and timelines for success have also varied. The meta-analysis reported a reduction in the mean UE rate from 6.5 to 4.4 UEs/100 intubation days.^4^ Others have reported a decrease in the UE rate below the benchmark of 1 per 100 intubated days.^2,13,17^ In a 54-bed level III NICU in France, the UE rate increased from 0.56 in the preintervention to 1.55 per 100 intubated days in the postintervention period over 2 years, despite measures to assess tube placement and position during every assessment and 2-person repositioning and retaping of the ET tube.^15^ The authors attributed the increase to discontinuation of tincture of benzoin, reduction of bilateral Y-shaped tape strips in tube fixation, and a high turnover of caregivers.^15^ Although we used several of the reported interventions, critical were the standardization of ET tube fixation and ongoing care.

The level IV NICU population is at high risk for UE because of referrals for chronic lung disease and evaluation for tracheostomy and prolonged ventilation. As in previous reports, nearly half of our UE events occurred in infants with prior extubation.^15,21^ The preponderance of UE events in infants larger than 2 kg was also probably a reflection of our level IV NICU population with more surgical infants needing prolonged intubation. The meta-analysis of UE in the NICU found that bradycardia after UE occurred in 39%−46% of infants, and cardiopulmonary resuscitation was required in 5%−13%.^4^ The rates of bradycardia in our study were lower, but desaturation occurred in half the cases. In the current study, we found that a quarter to a third of UE events did not require reintubation within 4 hours, suggesting (1) that a trial of elective extubation could have been attempted earlier and (2) that these infants did not suffer immediate apparent harm. Nonetheless, UE may cause infants who could otherwise have been stable and successfully extubated in optimal conditions to deteriorate acutely.

### Limitations

Although real-time audits of device use and 2-person handling were frequent, they were not consistently done or shared with staff. Although we made incremental changes through the QI process, compliance with specific interventions may have varied. Because of these caveats, we were unable to determine which interventions were most efficacious; an orchestrated testing approach in a multicenter QI may best answer this question. The baseline period of 5 months was somewhat short. Simultaneous QI projects targeted at improved postoperative handoffs and euthermia may have confounded our results. We suspect that the issues with constant training of nursing and trainee physician staff, turnover, and competing priorities are common to most level IV NICUs. However, other centers may have found it easier to implement practices and processes to achieve a more rapid reduction in UE rates and sustain over time. Despite these limitations, the findings of this long CQI project provide a roadmap for reducing UE rates in large academic units with complex patients.

## CONCLUSIONS

This CQI study at a regional level IV NICU achieved high compliance in practices and a staff communication model directed at a quality goal. Reduction in UE rate was achieved, albeit after 5 years. The challenge will be to sustain the changes in processes and training to achieve a stable reduction in UE, which is feasible in other units.

## DISCLOSURE

The authors have no financial interest to declare in relation to the content of this article.
